# The Dose–Response Effects of Vitamin D_3_ on Serum 25-Hydroxyvitamin D Levels in Vitamin D-Deficient Young Indian Women: A Randomized Controlled Trial

**DOI:** 10.3390/nu18091476

**Published:** 2026-05-06

**Authors:** Chandni Halcyon Peris, Sumithra Selvam, Sumathi Swaminathan, Ravi Rangarajan, Anura V. Kurpad, Tony Raj, Prashanth Thankachan

**Affiliations:** 1 Manipal Academy of Higher Education (Research Scholar), Manipal 576104, KA, India; chandni.hp@sjri.res.in; 2 Division of Nutrition, St. John’s Research Institute, St. John’s National Academy of Health Sciences (a Unit of CBCI Society for Medical Education), Bangalore 560034, KA, India; sumathi@sjri.res.in; 3 Division of Epidemiology, Biostatistics and Population Health, St. John’s Research Institute, St. John’s National Academy of Health Sciences (a Unit of CBCI Society for Medical Education), Bangalore 560034, KA, India; sumithrars@sjri.res.in; 4 Department of Environmental Health and Safety, University of Doha for Science and Technology, Doha 24449, Qatar; ravi.rangarajan@udst.edu.qa; 5 Department of Physiology, St. John’s Medical College, St. John’s National Academy of Health Sciences (a Unit of CBCI Society for Medical Education), Bangalore 560034, KA, India; a.kurpad@sjri.res.in (A.V.K.); tonyraj@sjri.res.in (T.R.); 6 Division of Medical Informatics, St. John’s Research Institute, St. John’s National Academy of Health Sciences (a Unit of CBCI Society for Medical Education), Bangalore 560034, KA, India

**Keywords:** vitamin D deficiency, vitamin D_3_ dietary intake, 25-hydroxyvitamin D, bone turnover markers, parathyroid hormone, bone-specific alkaline phosphatase, osteocalcin, dose–response, randomized controlled trial

## Abstract

**Background/Objectives**: Vitamin D deficiency is highly prevalent among young Indian women. This study evaluated the dose–response effects of varying levels of daily oral vitamin D_3_ intake on serum 25-hydroxyvitamin D [25(OH)D] concentrations and related bone biomarkers in vitamin D-deficient and insufficient young women. **Methods**: In a 12-week, double-blind, randomized controlled trial conducted in urban Bangalore, 108 non-pregnant, non-lactating women aged 18–35 years with serum 25(OH)D <20 ng/mL were randomized (1:1:1:1) to receive daily vitamin D_3_ at doses of 0, 400, 600, or 800 IU delivered via a fortified chocolate wafer. Serum 25(OH)D, parathyroid hormone (PTH), bone-specific alkaline phosphatase (BSAP), and osteocalcin were measured at baseline, and at weeks 4, 8, and 12. Intention-to-treat analysis was performed using mixed linear and logistic models to assess the effect of intervention. **Results:** At baseline, 76.4% of participants were vitamin D-deficient (<12 ng/mL) and 23.6% were insufficient (12–20 ng/mL). Serum 25(OH)D increased significantly over time in the 400, 600, and 800 IU/day groups, with a significant time × dose interaction effect (*p* < 0.001), demonstrating an overall graded response across doses. By week 12, vitamin D sufficiency (≥20 ng/mL) was achieved in 7.4%, 26.9%, 37.0%, and 65.4% of participants in the 0, 400, 600, and 800 IU groups, respectively. From baseline to week 12, the mean increase in serum 25(OH)D was 7.69 ng/mL in the 400 IU group, 8.83 ng/mL in the 600 IU group, and 10.23 ng/mL in the 800 IU group; no significant difference was observed between the 400 IU and 600 IU groups, whereas the 800 IU group demonstrated the greatest overall increase. PTH decreased over time without a significant time × dose interaction. No significant interaction effect was noted for BSAP and osteocalcin. Mean compliance was high (>98%) across all groups, and no serious adverse events were reported. **Conclusions:** Daily dietary intake of vitamin D_3_ at doses of 400–800 IU for 12 weeks significantly improved serum 25(OH)D concentrations in vitamin D-deficient young Indian women. While PTH levels decreased over time, effects on bone turnover markers were modest and not dose specific. A daily dose of 800 IU/day was the most effective, achieving vitamin D sufficiency in 65% of participants.

## 1. Introduction

Vitamin D deficiency is one of the most prevalent micronutrient deficiencies worldwide, affecting over one billion people [1]. India reports some of the highest rates of vitamin D deficiency globally, with prevalence estimates ranging from 70% to nearly 100% across diverse populations in both urban and rural settings [2]. This widespread deficiency is attributed to several contributing factors, including limited skin exposure to sunlight due to cultural clothing practices and predominantly indoor lifestyles, high levels of atmospheric pollution, and darker skin pigmentation, which reduces the efficiency of cutaneous vitamin D synthesis [3,4,5]. In the Indian context, dietary intake of vitamin D is negligible, and fortification of staple foods such as milk, flour, and edible oils has only recently been operationalized, following the formal notification in 2018 issued by Food Safety and Standards Authority of India [6].

As a fat-soluble secosteroid hormone, vitamin D plays a critical role in skeletal development and bone remodeling and is essential for maintaining calcium and phosphorus homeostasis [7,8]. The biological actions of vitamin D are mediated by its active form, 1,25-dihydroxyvitamin D, which binds to vitamin D receptors (VDRs) in various tissues throughout the body to regulate calcium absorption, bone remodeling and other cellular functions [9]. Serum 25-hydroxyvitamin D [25(OH)D] is the primary biomarker used to assess vitamin D status; according to the Institute of Medicine (IOM), concentrations <12 ng/mL indicate deficiency, 12–20 ng/mL indicate insufficiency, and ≥20 ng/mL are considered sufficient [10,11]. Serum 25(OH)D is increasingly recognized as a clinically relevant nutritional biomarker that reflects the integrated effects of dietary intake, supplementation, and endogenous synthesis, thereby informing individualized nutritional assessment and intervention strategies. Deficiency during childhood, adolescence, and early adulthood—critical periods for peak bone mass accrual—can impair bone mineralization, increase secondary hyperparathyroidism, and elevate long-term risk of osteoporosis and fractures [12]. Suboptimal status in women of reproductive age may prevent attainment of optimal peak bone mass, a key determinant of lifelong skeletal health [13]. Low 25(OH)D has also been associated with increased risk of infections, cardiometabolic disorders, autoimmune conditions, and adverse pregnancy outcomes, although causality remains under investigation [1,8,14]. Adequate vitamin D status depends on sufficient cutaneous synthesis and/or dietary intake across the life course.

The current Indian dietary allowances recommend an Estimated Average Requirement (EAR) of 400 IU/day and the Recommended Dietary Allowance (RDA) of 600 IU/day for vitamin D and emphasize the importance of regular sun exposure and outdoor physical activity to help maintain adequate vitamin D status [15]. However, dietary intake data indicate that actual vitamin D consumption remains well below these recommendations, particularly among low-income, urban women with limited sun exposure, predominantly on vegetarian diets, and with poor access to fortified foods [2]. In our earlier cross-sectional study among young urban Indian women, mean dietary vitamin D intake was approximately 45–50 IU/day, far below the recommendation, underscoring the negligible contribution of vitamin D from habitual diets in this population [16].

Targeted interventions are needed to address the long-term health consequences of vitamin D deficiency in vulnerable populations. Randomized controlled trials have demonstrated a dose–response relationship between oral vitamin D intake and circulating serum 25(OH)D concentrations, with diminishing incremental increases observed at higher intake levels [17,18,19]. However, the magnitude of the 25(OH)D response to supplementation varies substantially between individuals and is influenced by baseline vitamin D status, body mass index, adherence, and genetic factors [20]. Despite the growing body of evidence from dose–response trials, most studies have been conducted in high-income populations, with relatively limited representation of Asian populations, particularly women of reproductive age [18,21,22].

To address this evidence gap, we conducted a 12-week, double-blind, randomized controlled trial to evaluate the dose–response effects of daily oral vitamin D_3_ intake at 0 IU, 400 IU, 600 IU, and 800 IU in non-pregnant, non-lactating, vitamin D-deficient and insufficient Indian women aged 18–35 years living in urban Bangalore, India. The vitamin D doses selected, correspond to the current EAR (400 IU/day) for healthy populations and two incremental intake levels (600 IU/day and 800 IU/day) to explore if these doses are enough to move from a state of deficiency to sufficiency using a food-based approach.

The primary objective of the current study was to assess the dose–response effects of daily vitamin D_3_ intake at 0, 400, 600, and 800 IU on serum 25-hydroxyvitamin D [25(OH)D] concentrations and to determine the proportion of participants achieving vitamin D sufficiency (≥20 ng/mL) over a 12-week intervention period.

## 2. Materials and Methods

### 2.1. Study Design

This double-blind, randomized controlled trial was conducted at the St. John’s Research Institute, St. John’s National Academy of Health Sciences, Bangalore, India, to evaluate the dose–response effects of daily vitamin D_3_ intake of 0 IU, 400 IU, 600 IU, and 800 IU delivered via a fortified chocolate wafer product.

Non-pregnant, non-lactating women with vitamin D insufficiency/deficiency, defined as serum 25-hydroxyvitamin D concentrations <20 ng/mL, were randomized into one of four intervention groups corresponding to the assigned daily vitamin D_3_ dose (0, 400, 600 and 800 IU). The intervention period lasted 12 weeks, with data collected at four scheduled time points: baseline (week 0), week 4, week 8, and endline (week 12). The study protocol was approved by the Institutional Ethics Committee of St. John’s Medical College and Hospital (IEC Ref. No: 370/2018) and registered with the Clinical Trials Registry of India (CTRI/2020/03/024395). Written informed consent was obtained from all participants prior to enrolment.

### 2.2. Sample Size Estimation

Sample size was estimated using serum 25(OH)D concentration as the primary outcome. Based on previously published randomized vitamin D supplementation studies conducted in adult populations [23,24], baseline serum 25(OH)D concentrations of approximately 53 nmol/L (21.2 ng/mL) and post-supplementation concentrations of approximately 82 nmol/L (32.8 ng/mL) were considered. Studies conducted exclusively in elderly populations or in vitamin D–insufficient cohorts were excluded to better reflect the target population. The difference between baseline and post-supplementation concentrations (29 nmol/L [11.6 ng/mL]) represents the expected increase in serum 25(OH)D following vitamin D supplementation and was used to define the anticipated contrast between the supplemented groups and the placebo group for sample size estimation.

A conservative standard deviation of 30 nmol/L was assumed to account for inter-individual variability in vitamin D response. To detect this difference with 80% power at a two-sided significance level of 0.05, adjustment for multiple comparisons was applied using the Bonferroni correction for three primary contrasts (0 IU/day vs. 400 IU/day, 600 IU/day, and 800 IU/day). While a strict Bonferroni correction corresponds to an adjusted α of 0.0167, an α of 0.02 was used during the sample size planning stage as an approximation of the Bonferroni adjusted threshold. Under these assumptions, the required sample size was 23 participants per group. Allowing an anticipated 15% attrition, 27 participants were recruited per group, yielding a total sample size of 108 participants.

### 2.3. Recruitment of Participants

Screening for eligible study participants was conducted between August and December 2021, during which blood samples were collected for assessment of 25(OH)D status. Vitamin D status was classified according to criteria proposed by the Institute of Medicine (IOM, 2011), with serum 25(OH)D concentrations <12 ng/mL indicating deficiency, 12–20 ng/mL indicating insufficiency (risk of inadequacy) and ≥20 ng/mL considered sufficient for bone health [11].

Women with serum 25(OH)D concentrations <20 ng/mL and body mass index (BMI) within the eligible range, were enrolled and randomly allocated in equal proportions to one of four intervention groups (0 IU, 400 IU, 600 IU, or 800 IU/day of vitamin D_3_). Participants were excluded if they had consumed vitamin D or calcium supplements within the past month, a period considered sufficient to minimize residual effects of prior supplementation on circulating 25(OH)D concentrations given its biological half-life; had a history of chronic illnesses including diabetes, liver or kidney disease, cardiovascular disease, or gastrointestinal disorders affecting nutrient absorption; had hypo or hyperparathyroidism; were on medications known to influence vitamin D or calcium metabolism; or had participated in another clinical trial within the preceding three months.

### 2.4. Randomization and Blinding

Participants were randomly assigned in a 1:1:1:1 ratio to receive 0 IU, 400 IU, 600 IU, or 800 IU of vitamin D_3_ daily using a computer-generated sequence. Allocation was concealed using colour-coded labels linked to dose groups, with the blinding key maintained by an independent individual not involved in study procedures. The intervention products were identical in appearance and packaging and were dispensed in sealed packets every two weeks. Participants, investigators, and laboratory personnel remained blinded to group allocation until completion of the final data analysis.

### 2.5. Study Intervention

#### 2.5.1. Product Composition

The intervention consisted of a daily vitamin D_3_–fortified chocolate wafer administered for 12 weeks at four dose levels: 0 IU (placebo), 400 IU, 600 IU, and 800 IU of cholecalciferol. Participants consumed one wafer per day, which served as the sole delivery vehicle for the assigned dose. Each wafer comprised a plain wafer base layered with dark compound chocolate and the allocated vitamin D_3_ dose, forming a uniform and palatable delivery vehicle.

Vitamin D_3_ was incorporated using pharmaceutical-grade cholecalciferol granules (D-Rise sachets, USV Pvt Ltd., Mumbai, India; 1 g = 60,000 IU). Individual doses corresponding to 400 IU, 600 IU, and 800 IU were prepared by accurately weighing 6.7 mg, 10.0 mg, and 13.3 mg of granules, respectively, using a calibrated analytical balance (Sartorius Research Series, Model R200D; readability 0.00001 g). Plain wafer sheets (Vivinda Plain Wafers, Raj Classic Foods Ltd., Hyderabad, India) and dark compound chocolate chips (PAI^®®^, Soubhagya Confectionery Pvt Ltd., Hyderabad, India) were procured in bulk from single batches prior to study initiation to ensure consistency in composition, appearance, and shelf life. Wafers were selected due to their uniform size, ease of handling, and high participant acceptability.

#### 2.5.2. Product Preparation and Packaging

Intervention wafers were prepared weekly by trained laboratory staff in the clean metabolic kitchen at St. John’s Research Institute, Bangalore, India, following a predefined Standard Operating Procedure routinely used for food-based intervention studies at the institute. The allocated vitamin D_3_ dose was placed between two wafer layers, with melted dark compound chocolate used solely as an adhesive medium. Wafers were assembled under standardized indoor conditions, individually wrapped in aluminium foil, and packaged into sealed pouches (15 wafers per pouch). To minimize inter-batch variability and ensure freshness, wafers were distributed for consumption within 1–2 weeks of preparation.

Identical placebo wafers (0 IU) were prepared using the same procedure without the addition of vitamin D_3_ and were visually indistinguishable from fortified wafers to maintain blinding. Participants were instructed to consume one wafer daily, not to compensate for missed doses, and not to share wafers. Compliance was monitored through regular telephone follow-up and collection of returned wafers during follow-up visits.

#### 2.5.3. Nutrient Analysis and Dose Verification

Macronutrient composition and vitamin D_3_ content of the intervention wafers were assessed by a NABL and FSSAI accredited laboratory. Representative samples from independently prepared batches were analysed to verify nutrient composition and dose accuracy. Mean vitamin D_3_ recoveries were 437.5 IU, 639.2 IU, and 792.8 IU for the target doses of 400 IU, 600 IU, and 800 IU, respectively. Each wafer provided approximately 5.9 kcal of energy, with minimal contributions from macronutrients, and was designed as a low-calorie delivery vehicle to avoid confounding habitual dietary intake.

### 2.6. Compliance Monitoring

Compliance with the intervention was monitored weekly throughout the 12-week study using a structured daily self-report checklist. Participants recorded daily consumption of the study wafer using tick (✓) marks for intake and cross (×) marks for missed doses. Completed compliance forms were reviewed at each scheduled visit, and missed or extra doses, along with reasons for non-compliance, were documented by study staff. Adherence was reinforced through periodic follow-up telephone calls during the intervention period.

### 2.7. Anthropometry

Weight and height were measured using a calibrated digital scale (ESSAE DS-415) and a portable stadiometer (HM-01, Standard Steel, Haryana, India), respectively, to the nearest 0.1 kg and 0.1 cm. Height was assessed once at baseline, while weight was recorded at baseline and at weeks 4, 8, and 12 during scheduled clinic visits. All measurements were conducted in the morning after an overnight fast (8–10 h), with participants wearing light clothing and no footwear or accessories to ensure accuracy. Body mass index (BMI) was calculated as weight (kg) divided by height in meters squared (m^2^) [25]. Only women with a BMI within the normal range (18.5–24.9 kg/m^2^; World Health Organization [25]) were considered eligible for participation and were included in the study intervention.

### 2.8. Biochemical Assessments

Fasting venous blood samples were collected in the morning after an overnight fast (8–10 h) at four timepoints: baseline (week 0), week 4, week 8, and endline (week 12). Fasting was maintained to ensure consistency across analytes, particularly for parathyroid hormone (PTH) and osteocalcin, which are sensitive to recent food intake and exhibit circadian variation [26]. Venous blood (8 mL) was drawn under aseptic conditions and processed within 30 min of collection. Samples were centrifuged at 3000 rpm for 15 min at 4 °C to separate serum, aliquoted and stored at −80 °C until biochemical analysis. Serum 25(OH)D was measured for all screened participants, while PTH, osteocalcin, and bone-specific alkaline phosphatase (BSAP) were estimated at all four timepoints only in participants recruited into the trial (*n* = 108). The 25(OH)D, PTH, and osteocalcin were measured using electrochemiluminescence immunoassay on the Roche Cobas E411 (Roche Diagnostics, GmbH, Mannheim, Germany), and BSAP was analyzed using an immunoenzymatic assay (Ostase BAP EIA kit, Immunodiagnostic Systems Ltd., Boldon, Tyne and Wear, UK) and concentrations in the samples were measured at 405 nm using a microplate reader (AM 2100, Alere™, Abbott Laboratories, Abbott Park, IL, USA). Intra and inter-assay coefficients of variation were <5% for all analytes. Based on the IOM guidelines, the 25(OH)D status was classified as: deficiency (<12 ng/mL), insufficiency (12–20 ng/mL), and sufficiency (≥20 ng/mL) [11]. For the remaining biomarkers, reference ranges were defined as: 16–87 pg/mL for PTH, 9–40 ng/mL for osteocalcin, and 0–14 μg/L for BSAP in premenopausal women [27].

### 2.9. Dietary Intake Assessment

Dietary intake was assessed at baseline and endline using three consecutive 24 h dietary recalls (two weekdays, one weekend), collected within a single week at each time point. Interviews were conducted by trained nutritionists using visual aids and standardized utensils to estimate portion sizes. Nutrient intakes for each individual were computed from standardized recipes using raw food values from the Indian Food Composition Tables, and for those nutrients for which values were not available, from the USDA food composition database [28]. For each participant, the mean daily intake across the three recall days was computed and used for statistical analysis. Mean daily intakes of energy, macronutrients (protein, fat, carbohydrate), and key micronutrients (calcium, phosphorus, and iron) were calculated [15].

### 2.10. Sun Exposure Assessment and Morbidity Surveillance

Sun exposure was assessed at baseline and endline using a structured questionnaire adapted from previously validated tools used in Indian settings [29,30]. The questionnaire collected information on average daily duration of exposure (minutes/day), time of day, use of sunscreen including sun protection factor (SPF), and sun-protective practices such as the use of scarves, umbrellas, or sunglasses. Responses were used to categorize habitual sun exposure during the preceding month. These variables were summarized descriptively to characterize habitual sun exposure behaviours during the study period and to assess whether these behaviours remained stable over the intervention; they were not included as covariates in the primary statistical analyses.

Participants self-reported the occurrence of common symptoms, including diarrhoea, abdominal pain, fever, sore throat, cough, respiratory discomfort, and reduced food intake, and indicated the days of symptom occurrence (Monday to Sunday). Study staff additionally documented whether medical care was sought, hospitalization occurred, or self-medication was used.

### 2.11. Statistical Analysis

Descriptive statistics are reported as means and standard deviations (SD) for normally distributed data and as median (quartile 1, quartile 3) for non-normally distributed variables, categorical variable are expressed as frequencies and percentages. Sun exposure behaviours (duration, time of day, sunscreen use, and clothing coverage) were summarized descriptively using frequencies and percentages. Baseline characteristics between study groups are compared using one-way analysis of variance (ANOVA) for continuous variables and Chi-square tests for categorical variables. In addition, multiple comparisons were performed using Bonferroni correction. Intention to treat analysis was performed using mixed linear and logistic models to assess the changes in the outcome, (for serum 25(OH)D, PTH, BSAP, and osteocalcin levels) over time [at baseline, week 4, week 8, and week 12], accounting for within-subject correlation due to repeated measurements (baseline and follow-up measurements) allowing estimation of longitudinal change trajectories over time. Fixed effects included vitamin D3 dose treatment group, time (categorical), and a group-by-time interaction, allowing estimation of differential change trajectories between groups. A random intercept of individuals was included to account for within-subject correlation. By incorporating baseline measurements within the repeated-measures structure, the linear mixed-effects model accounted for baseline differences and estimated longitudinal change trajectories over time. Categorical variables representing vitamin D status (deficient, insufficient, sufficient) were compared across dose groups using Pearson’s Chi-square test. For dietary intake data, between-group comparisons were performed using the Kruskal–Wallis test, and within-group changes from baseline to endline were assessed using the Wilcoxon signed-rank test. A *p* value < 0.05 (two-sided) was considered statistically significant. All statistical analyses were performed using IBM SPSS Statistics, version 29.0 (IBM Corp., Armonk, NY, USA).

## 3. Results

### 3.1. Participant Baseline Characteristics

**Figure 1:**
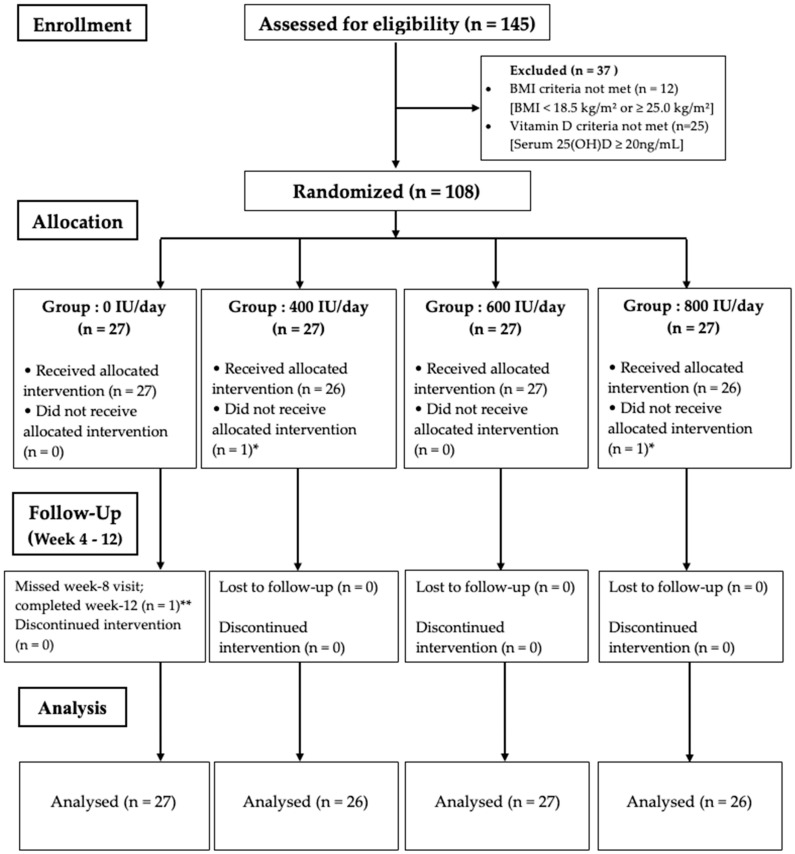
CONSORT flow diagram of participant recruitment, allocation, follow-up, and analysis. A total of 108 participants were randomized. * One participant each in the 400 IU/day and 800 IU/day groups withdrew from the study after baseline measurements and did not receive the allocated intervention. ** One participant in the 0 IU/day group missed the week 8 visit but completed the week 12 assessment. 25(OH)D: 25-hydroxyvitamin D.

A total of 108 participants were randomized to receive daily oral vitamin D_3_ at doses of 0 IU, 400 IU, 600 IU, or 800 IU for 12 weeks (*n* = 27 per group) (Figure 1). Two participants (one each in the 400 IU and 800 IU groups) withdrew after baseline screening but before initiation of the intervention and therefore did not consume the study product or contribute follow-up outcome data. One participant in the 0 IU group missed the week 8 blood draw but completed all other assessments. For dietary intake analyses, one participant each in the 400 IU and 800 IU groups had incomplete paired baseline and endline dietary recall data; therefore, dietary analyses for these groups were conducted in *n* = 26 participants.

Baseline demographic, anthropometric, and biochemical characteristics were comparable across groups, with no statistically significant between-group differences (*p* > 0.05), except for serum 25(OH)D, where the 0 IU group had a slightly lower mean concentration compared with the 800 IU group (*p* = 0.029). However, analyses were conducted using repeated measures over time, and the significant time × group interaction indicates that the observed differences reflect treatment effects rather than baseline imbalance. Furthermore, when classified categorically, baseline vitamin D status (deficient vs. insufficient) did not differ significantly between groups (Table 1). The mean age of enrolled participants was 20.9 ± 1.1 years, mean BMI was 21.2 ± 1.9 kg/m^2^, and 76.4% were vitamin D-deficient (<12 ng/mL) while 23.6% were insufficient (12–20 ng/mL); no participants were sufficient at baseline.

### 3.2. Change in Vitamin D Status and Serum Biomarkers by Timepoint and Dose Group

At baseline, all participants were classified as either vitamin D-deficient or insufficient, and none were sufficient (Table 2). Significant between-group differences in vitamin D status were observed across timepoints (Chi-square *p* < 0.001). By week 12, 65.4% of participants in the 800 IU group, 37.0% in the 600 IU group, and 26.9% in the 400 IU group became vitamin D sufficient, whereas only 7.4% in the 0 IU group were sufficient (Table 2); these differences occurred despite comparable changes in dietary intake and sun exposure across groups.

**Figure 2:**
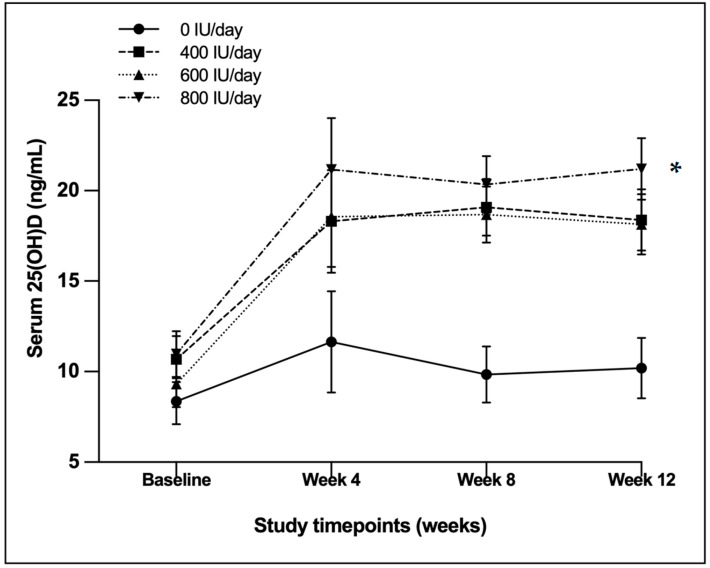
Serum 25-hydroxyvitamin D [25(OH)D] (ng/mL) concentrations over 12 weeks by vitamin D_3_ dose group (0, 400, 600, and 800 IU/day). Values are presented as means with 95% confidence intervals. * At week 12, the 400, 600, and 800 IU/day groups were significantly different from the control group, with no significant difference between the 400 and 600 IU/day groups. Time effect, *p* < 0.001, Interaction effect, *p* < 0.001.

To account for the initial differences in the serum 25(OH)D concentrations, baseline values were incorporated within the repeated-measures structure of the linear mixed effects model; serum 25(OH)D concentrations increased significantly over time in all supplemented groups, with a significant time effect (*p* < 0.001) and time × group interaction (*p* < 0.001) (Table 3; Figure 2). Relative to baseline, mean 25(OH)D increased by 7.69 ng/mL in the 400 IU group, 8.83 ng/mL in the 600 IU group, and 10.23 ng/mL in the 800 IU group; changes were not significant in the 0 IU group. No significant differences were observed between the 400 IU and 600 IU groups. Serum PTH decreased over time (time effect *p* = 0.003) but without a significant time × group interaction (*p* = 0.608). BSAP showed a significant time effect (*p* < 0.001) with a transient increase at week 4 followed by a decline toward week 12; no time × group interaction was observed (*p* = 0.423). Osteocalcin exhibited a significant change over time when analysed across all groups (*p* < 0.001), characterized by a transient decline at week 4 followed by partial recovery by weeks 8 and 12. However, the absence of a significant time × group interaction (*p* = 0.235) indicates that this temporal pattern was consistent across all dose groups, including placebo, and therefore unlikely to reflect a dose-dependent effect of vitamin D_3_ supplementation.

Based on estimated marginal means (EMMs), serum 25(OH)D concentrations increased across the intervention groups, with week 12 values of 18.38 ng/mL (95% CI: 16.46–20.30), 18.14 ng/mL (16.25–20.00), and 21.20 ng/mL (19.28–23.10) in the 400, 600, and 800 IU/day groups, respectively. Week 12 values were similar between the 400 and 600 IU/day groups, with higher values observed in the 800 IU/day group; however, no statistically significant differences were observed between dose groups at week 12. PTH concentrations decreased over time, with week 12 EMMs of 40.20 pg/mL (33.60–46.70), 43.20 pg/mL (36.80–49.70), and 43.70 pg/mL (37.20–50.30) in the 400, 600, and 800 IU/day groups, respectively. BSAP concentrations increased initially and then declined by week 12, with week 12 EMMs of 29.30 U/L (26.10–32.60), 28.20 U/L (25.00–31.40), and 28.40 U/L (25.10–31.70) in the 400, 600, and 800 IU/day groups, respectively. Osteocalcin showed variable temporal changes, with week 12 EMMs of 19.80 ng/mL (16.20–23.30), 24.50 ng/mL (21.00–27.90), and 24.60 ng/mL (21.00–28.10) in the 400, 600, and 800 IU/day groups, respectively.

### 3.3. Dietary Intake

Energy, carbohydrate, fat, and calcium intakes increased significantly from baseline to endline in all dose groups (Wilcoxon signed-rank test, *p* < 0.05), with no significant between-group differences at either time point (Kruskal–Wallis test, *p* > 0.05). Mean calcium intake at endline ranged from 619.7 ± 182.6 mg/day (600 IU) to 633.3 ± 211.2 mg/day (800 IU), while mean phosphorus intake ranged from 1205.6 ± 267.9 mg/day (0 IU) to 1283.2 ± 269.4 mg/day (400 IU) (Table A1). Among food groups (Table A1), intakes of fats and oils, sugar and sweets, milk and milk products, and eggs showed significant within-group increases, whereas legume and pulse intake showed a modest decline. Intakes of vegetables, roots and tubers, and meat and poultry remained largely unchanged throughout the intervention period.

Compliance with the intervention was high across all groups, with mean compliance of 98.6%, 98.8%, 96.7%, and 99.3% in the 0 IU, 400 IU, 600 IU, and 800 IU groups, respectively. No serious adverse events or health complaints were reported during the 12-week study period.

### 3.4. Sun Exposure

When data were pooled across study arms, 76.4% of participants reported ≤15 min of daily sun exposure at baseline (25.5% <5 min; 50.9% 5–15 min), while 23.6% reported >15 min. At 12 weeks, 70.8% continued to report ≤15 min of exposure (24.5% <5 min; 46.2% 5–15 min), whereas 29.2% reported >15 min, indicating persistently low overall sun exposure during the study period. Regular sunscreen use remained low (<10%).

## 4. Discussion

This randomized controlled trial evaluated the dose–response effects of daily oral vitamin D_3_ intake (0, 400, 600, and 800 IU/day) over 12 weeks in young Indian women with vitamin D deficiency. The increase in serum 25(OH)D across supplemented groups, together with the clear gradient in the proportion of participants achieving vitamin D sufficiency by week 12, supports the conclusion that 800 IU/day was the most effective among the tested doses. The intervention showed an overall graded response across doses, with the clearest clinical advantage observed at 800 IU/day. The primary outcome, serum 25(OH)D, demonstrated an overall graded increase across dose groups over time, accompanied by a reduction in PTH levels, whereas secondary outcomes (BSAP and osteocalcin) showed time-related changes without evidence of dose-specific effects.

Serum 25(OH)D concentrations increased significantly over time in all supplemented groups, with a significant time effect and time × group interaction, confirming a dose-dependent response to supplementation. By week 12, 65.4% of participants in the 800 IU group achieved vitamin D sufficiency (≥20 ng/mL), compared with 37.0% and 26.9% in the 600 IU and 400 IU groups, respectively, and only 7.4% in the placebo group. These findings support prior dose–response trials that reported similar improvements with daily intakes of 600–800 IU/day, particularly among deficient populations [18,31]. The significant time × group interaction observed for 25(OH)D levels further confirms the efficacy of intervention in a dose-specific manner. Notably, even the 400 IU dose (corresponding to Indian EAR) [15] led to substantial improvements by week 4, with 23.1% of participants achieving vitamin D sufficiency, although fewer participants reached sufficiency compared with the higher dose groups. While 400 IU/day was effective in eliminating deficiency (<12 ng/mL), higher doses (600–800 IU/day) were more consistently associated with attainment of vitamin D sufficiency (≥20 ng/mL) from a state of deficiency. In this context, serum 25(OH)D serves as a robust biochemical marker of vitamin D status, reflecting the cumulative effects of dietary intake and endogenous synthesis. Emerging perspectives in precision nutrition further emphasize the role of such biomarkers in guiding individualized nutritional strategies, where biochemical responses may provide a more accurate basis for intervention than intake estimates alone [32].

These findings are consistent with previous studies evaluating vitamin D supplementation kinetics across different dosing regimens [33,34,35], demonstrating that vitamin D supplementation, whether administered as daily, bolus, or intramuscular dosing, results in significant increases in serum 25(OH)D concentrations, with differences in response kinetics depending on dose, frequency, and route of administration. While high-dose regimens have been shown to achieve rapid normalization of 25(OH)D levels, our findings demonstrate that nutritionally relevant daily doses result in a gradual and sustained improvement in vitamin D status.

Although an inverse association between serum 25(OH)D and PTH was observed over the intervention period, the absence of a significant time × group interaction indicates that reductions in PTH were not dose dependent. This suggests that the observed decline in PTH reflects a general temporal response rather than a specific effect of increasing vitamin D_3_ intake. Such a pattern may indicate a physiological threshold beyond which further suppression of PTH is limited, particularly in young, otherwise healthy women without overt hyperparathyroidism or high baseline bone turnover [36]. Previous studies have reported that PTH suppression tends to plateau once serum 25(OH)D concentrations reach approximately 20–30 ng/mL, a range not consistently achieved across all dose groups or timepoints in the present study [37]. These findings are consistent with evidence from randomized, double-blind, placebo-controlled dose–response trials in vitamin D–insufficient women, where daily vitamin D_3_ intakes between 400 and 2400 IU increased serum 25(OH)D concentrations but did not produce significant dose-dependent changes in PTH [38].

Bone-specific alkaline phosphatase (BSAP) concentrations exhibited a significant change over time across all groups, characterized by a transient increase at week 4 followed by a gradual decline toward baseline by week 12. Importantly, this pattern was observed in all intervention groups, including placebo, and the absence of a significant time × group interaction indicates that these changes were not dose dependent. This temporal pattern is therefore unlikely to reflect a specific effect of vitamin D_3_ supplementation and may instead represent short-term physiological or adaptive variation. Evidence from randomized controlled trials and systematic reviews suggests that vitamin D supplementation alone, particularly at nutritionally relevant doses and in the absence of concurrent calcium intake, has inconsistent and generally modest effects on bone turnover markers such as BSAP [39,40]. Osteocalcin concentrations demonstrated a significant temporal change across the intervention period, characterized by an initial decline at week 4 followed by partial recovery by weeks 8 and 12. This pattern was observed across all dose groups, and the absence of a significant time × group interaction indicates that these changes were not dose dependent. While minor between-group differences in absolute values were observed at individual timepoints, these did not translate into a consistent or statistically significant dose-related effect of vitamin D_3_ supplementation. Consistent with previous randomized trials and systematic reviews, vitamin D supplementation alone has not been shown to exert a consistent effect on osteocalcin or other bone turnover markers unless administered at higher pharmacological doses, over longer durations, or in combination with calcium [40,41]. The absence of significant group effects suggests that bone marker responses were modest and variable over the study period, which may partly reflect the absence of concurrent calcium co-supplementation.

These findings align with the Daily D Health Study [42], in which racially diverse at-risk schoolchildren receiving 600, 1000, or 2000 IU/day vitamin D_3_ for six months exhibited dose-dependent increases in serum 25(OH)D, with attainment of sufficiency (defined as ≥30 ng/mL) occurring most consistently in the highest-dose (2000 IU/day) group. Importantly, the study also reported no significant changes over time or between dose groups in PTH or alkaline phosphatase, consistent with our observation that PTH declined over time but without a significant time × group interaction, and that BSAP and osteocalcin showed transient temporal fluctuations that were not dose dependent.

The absence of a dose-specific response in bone turnover markers, despite clear improvements in serum 25(OH)D, likely reflects the temporal dynamics of skeletal adaptation. In young, otherwise healthy women without overt bone disease, short-term vitamin D repletion is expected to restore circulating 25(OH)D concentrations before producing measurable downstream changes in bone turnover markers. Given the slower dynamics of skeletal remodeling, changes in PTH, BSAP, and osteocalcin may require longer durations of sustained vitamin D sufficiency. Thus, the observed pattern of improvement in vitamin D status without corresponding changes in skeletal markers is biologically plausible and should be interpreted with caution.

Additionally, in the same study, an apparent decline in mean 25(OH)D at a later follow-up after the supplementation period, despite concentrations remaining above baseline was observed, highlighting that improvements in vitamin D status may not be fully sustained without continued intake—an interpretation that supports the need for ongoing strategies (e.g., sustained fortification or supplementation) in high-risk settings.

Over our 12-week intervention, participants also demonstrated modest but consistent improvements in dietary intake across all dose groups. Energy intake increased by approximately 250–300 kcal/day from baseline to endline, primarily driven by higher carbohydrate and fat consumption. Protein intake increased slightly, averaging ~56–62 g/day at endline, consistent with the predominantly vegetarian dietary patterns in this population. Calcium intake improved across all groups, rising from ~500–530 mg/day at baseline to ~620–640 mg/day at endline; however, intakes remained below the EAR of 800 mg/day for women of reproductive age, indicating persistent inadequacy. Phosphorus intake exceeded requirements at all timepoints, remaining above 1200 mg/day at endline. Food groups contributing to vitamin D intake, including milk, eggs, and fish, showed modest increases. Milk and milk product intake increased to ~200–215 g/day, while fish intake remained minimal, particularly in the 800 IU group (from 0 g to ~7 g/day).

These dietary changes were comparable across intervention arms and are likely attributable to increased health awareness and repeated dietary assessments during study follow-up rather than a direct effect of vitamin D intervention. Despite these modest improvements, dietary intake alone remained insufficient to meet the ICMR-recommended EAR for vitamin D (400 IU/day), reflecting the limited contribution of vitamin D-rich foods in typical Indian diets. Consistent with this, dietary intakes changed similarly across all intervention arms, including placebo, suggesting that the observed changes in serum 25(OH)D and bone biomarkers were primarily driven by the vitamin D_3_ intervention rather than dietary modification. Indian studies have shown that relatively prolonged midday sun exposure (e.g., >1 h per day) may be required to maintain serum 25(OH)D concentrations ≥50 nmol/L in urban populations [30]. In contrast, over 70% of participants in our study reported ≤15 min of daily sun exposure at both baseline and endline, a duration likely insufficient for meaningful endogenous vitamin D synthesis in this setting. Reported energy intakes were consistent with expected reference values for sedentary Indian women of similar body weight, and protein intakes were within or above recommended levels. In the context of normal BMI, these findings do not indicate clinical undernutrition. However, dietary intake was assessed using self-reported methods, which are subject to inherent limitations, including potential underreporting.

The fortified wafers were the primary source of vitamin D_3_ during the trial, as intake from other fortified foods in their habitual diets was negligible, and no participants reported taking additional calcium or vitamin D supplements during the intervention period. By the end of the 12-week intervention, the majority of participants in the 800 IU/day group and a lower proportion of those in the 600 IU/day and 400 IU/day groups achieved vitamin D sufficiency (≥20 ng/mL). Based on the observed rates of increase, it is likely that additional time beyond 12 weeks would have been required for most participants in the 400 IU/day and 600 IU/day groups to achieve sufficiency. These findings align with previous evidence demonstrating that vitamin D_3_ supplementation, often provided alongside calcium and phosphate for bone health, significantly increases serum 25(OH)D concentrations but may not elicit measurable changes in bone turnover markers over short durations, particularly in healthy populations and especially in the absence of adequate calcium intake [43]. Taken together, these findings reinforce the limited role of habitual diet in addressing vitamin D deficiency in Indian women and underscore the need for fortified food-based strategies for achieving meaningful improvements in vitamin D status.

The 12-week intervention duration was chosen to capture meaningful dose–response changes in circulating 25(OH)D concentrations while remaining feasible within a controlled diet-based intervention framework. Evidence from other studies indicate that serum 25(OH)D approaches a near steady state within approximately 8–12 weeks of daily vitamin D_3_ intake, particularly at nutritionally relevant intake levels, making the time frame used in this study appropriate for evaluating biochemical repletion and attainment of vitamin D sufficiency. In contrast, structural skeletal changes and sustained alterations in bone turnover markers often require longer intervention periods and, in some cases, concurrent calcium supplementation coupled with physical activity. Accordingly, the modest and variable responses observed for bone turnover markers in the present study should be interpreted with caution given the intervention duration and the young, otherwise healthy study population.

### Strengths and Limitations

This study’s strengths include (a) double-blind randomized design, (b) use of a food vehicle (wafer), (c) vitamin D doses studied that were relevant from a dietary intake perspective, and (d) inclusion of a relatively homogeneous population of young women with vitamin D deficiency. Compliance with the intervention was good, and the use of repeated measures at different time points enhanced the reliability of within-subject comparisons.

However, this study does have limitations. The study population was restricted to young women with normal BMI, which may limit the generalizability of the findings to other populations, including men, adolescents, older adults, and individuals with overweight or obesity. The 12-week intervention duration, while sufficient to assess short-term changes in serum 25(OH)D concentrations, may not have been adequate to detect structural bone changes or longer-term skeletal adaptations. Although sun exposure and dietary intake were assessed, these variables were not incorporated into the primary analytical models. Physical activity levels were not formally assessed, and therefore energy requirements could not be individualized; however, the study population predominantly comprised students and young professionals with largely sedentary lifestyles. While these factors remained relatively stable over the study period, unmeasured environmental and behavioral influences may have contributed to inter-individual variability in response to supplementation. In addition, baseline dietary recalls were partially conducted telephonically during COVID-19 restrictions, whereas endline assessments were conducted entirely face-to-face; improved probing and portion-size estimation during the latter may have contributed to higher reported intakes.

Finally, biochemical markers of calcium homeostasis (such as serum calcium, phosphorus, and indicators of hypercalcemia), as well as broader markers of nutritional status (e.g., HbA1c, lipid profile, plasma proteins), were not assessed, as these were beyond the scope of the study, which was designed to evaluate the dose–response effects of vitamin D_3_ supplementation.

## 5. Conclusions

Daily dietary vitamin D_3_ intake at doses of 400–800 IU for 12 weeks resulted in significant, dose-dependent increases in serum 25(OH)D concentrations in vitamin D-deficient and -insufficient young Indian women. Mean adjusted increases from baseline were 6.5 ng/mL with 400 IU/day, 6.0 ng/mL with 600 IU/day, and 8.4 ng/mL with 800 IU/day, with no significant change observed in the placebo group. By week 12, vitamin D sufficiency (≥20 ng/mL) was achieved in 65.4% of participants receiving 800 IU/day, compared with 37.0% and 26.9% in the 600 IU/day and 400 IU/day groups, respectively.

Serum PTH decreased over time, while bone turnover markers (BSAP and osteocalcin) exhibited modest, non-dose-dependent temporal changes. These findings indicate that nutritionally relevant daily intakes of vitamin D_3_, particularly 800 IU/day, are effective in improving vitamin D status in deficient populations but may be insufficient to elicit consistent changes in bone turnover over a short intervention period in the absence of concurrent calcium supplementation. Longer-term studies incorporating calcium co-fortification are warranted to evaluate sustained skeletal responses and to inform public health strategies aimed at improving vitamin D adequacy in young Indian women.

## Figures and Tables

**Table 1:** Baseline Characteristics of Enrolled Participants by Intervention Group (*n* = 108).

Characteristic	Vitamin D_3_ Dose (IU/day)				*p* Value
	0 (*n* = 27)	400 (*n* = 27)	600 (*n* = 27)	800 (*n* = 27)	
Demographic and Anthropometric					
Age (years)	21.07 ± 1.47	20.88 ± 1.15	20.77 ± 0.67	20.98 ± 0.97	0.766
Weight (kg)	53.06 ± 5.15	53.28 ± 6.02	54.01 ± 5.01	52.88 ± 6.82	0.898
Height (cm)	159.55 ± 4.90	158.19 ± 5.94	157.77 ± 3.99	158.05 ± 6.37	0.624
BMI (kg/m^2^)	20.83 ± 1.70	21.29 ± 2.17	21.70 ± 1.85	21.11 ± 1.75	0.385

Values are presented as mean ± standard deviation. Abbreviations: BMI = Body Mass Index; *p*-values are from one way ANOVA. A *p*-value < 0.05 was considered statistically significant. No statistically significant differences in baseline characteristics were observed between study groups (*p* > 0.05).

**Table 2:** Proportion of Participants with Vitamin D Deficiency, Insufficiency, and Sufficiency by Dose Group and Study Timepoint (*n*, %).

Timepoint	Vitamin D Status ^1^	Vitamin D_3_ Dose (IU/day)				*p*-Value
		0 (*n* = 27)	400 (*n* = 27)	600 (*n* = 27)	800 (*n* = 27)	
**Baseline**	Deficient	25 (92.6%)	19 (73.1%)	21 (77.8%)	16 (61.5%)	0.078
	Insufficient	2 (7.4%)	7 (26.9%)	6 (22.2%)	10 (38.5%)	
**Week 4**	Deficient	19 (70.4%)	1 (3.8%)	6 (22.2%)	1 (3.8%)	
	Insufficient	2 (7.4%)	19 (73.1%)	12 (44.4%)	15 (57.7%)	<0.001
	Sufficient	6 (22.2%)	6 (23.1%)	9 (33.3%)	10 (38.5%)	
**Week 8**	Deficient	18 (66.7%)	0 (0.0%)	2 (7.4%)	1 (3.8%)	
	Insufficient	8 (29.6%)	16 (61.5%)	16 (59.3%)	9 (34.6%)	<0.001
	Sufficient	0 (0.0%)	10 (38.5%)	9 (33.3%)	16 (61.5%)	
**Week 12**	Deficient	17 (63.0%)	0 (0.0%)	2 (7.4%)	0 (0.0%)	
	Insufficient	8 (29.6%)	19 (73.1%)	15 (55.6%)	9 (34.6%)	<0.001
	Sufficient	2 (7.4%)	7 (26.9%)	10 (37.0%)	17 (65.4%)	

^1^ Values are represented as number and percentage (*n*, %). Classification of vitamin D status was based on serum 25(OH)D concentration IOM cut-offs: deficient (<12 ng/mL), insufficient (12–20 ng/mL), and sufficient (≥20 ng/mL). Between-group comparisons made using Pearson’s Chi-square test. Abbreviations: IU = International Units.

**Table 3:** Descriptive statistics (mean ± SD) of serum 25-hydroxyvitamin D, parathyroid hormone, bone-specific alkaline phosphatase, and osteocalcin concentrations by vitamin D_3_ dose group across four timepoints (baseline, week 4, week 8, and week 12).

Biomarkers	Timepoint	Vitamin D_3_ Dose (IU/Day)				Time Effect (*p*)	Interaction Effect (*p*)
		0 (*n* = 27)	400 (*n* = 26)	600 (*n* = 27)	800 (*n* = 26)		
25(OH)D (ng/mL)	Baseline	8.37 ± 1.95 ^b^	10.69 ± 3.98 ^a^	9.31 ± 3.27 ^a^	10.97 ± 3.70 ^a^		
	Week 4	11.64 ± 6.39 ^a^	18.31 ± 6.75 ^b^	18.57 ± 7.87 ^b^	21.29 ± 8.11 ^b^		
	Week 8	9.98 ± 3.70 ^a^	19.09 ± 3.59 ^b^	18.69 ± 5.07 ^b^	20.43 ± 3.62 ^b^	<0.001	<0.001
	Week 12	10.19 ± 5.04 ^a^	18.39 ± 3.27 ^b^	18.14 ± 5.22 ^b^	21.27 ± 3.54 ^b^		
PTH (pg/mL)	Baseline	48.45 ± 22.75	43.46 ± 15.56	49.14 ± 16.20	50.28 ± 16.00		
	Week 4	54.28 ± 15.78	41.52 ± 15.49	48.32 ± 19.15	49.03 ± 22.40		
	Week 8	50.04 ± 16.05	41.13 ± 15.30	42.27 ± 17.93	42.91 ± 15.67	0.003	0.608
	Week 12	46.75 ± 16.88	40.30 ± 16.10	43.25 ± 12.78	43.61 ± 16.47		
BSAP (U/L)	Baseline	20.21 ± 7.39	18.02 ± 9.12	20.56 ± 11.34	22.07 ± 10.32		
	Week 4	30.83 ± 10.63	32.85 ± 9.16	30.82 ± 7.01	34.46 ± 7.17		
	Week 8	30.58 ± 8.47	33.46 ± 10.13	29.48 ± 8.18	32.15 ± 7.84	<0.001	0.423
	Week 12	29.30 ± 8.30	29.54 ± 6.85	28.19 ± 5.36	28.54 ± 5.43		
Osteocalcin (ng/mL)	Baseline	28.07 ± 9.20	24.61 ± 9.35	22.68 ± 8.42	24.72 ± 9.08		
	Week 4	20.90 ± 8.19	18.03 ± 8.94	16.74 ± 8.63	18.52 ± 9.76		
	Week 8	23.93 ± 9.06	21.79 ± 8.13	25.68 ± 9.99	26.70 ± 9.04	<0.001	0.235
	Week 12	22.11 ± 8.46	19.79 ± 10.23	24.45 ± 9.43	24.57 ± 10.56		

Data are presented as observed mean ± standard deviation (SD) at each study timepoint. Superscripts ^a,b^ for serum 25(OH)D denote statistically significant between-group differences across time based on pairwise comparisons of estimated marginal means with Bonferroni adjustment (*p* < 0.05); values sharing the same superscript are not significantly different. *p*-values for the main time effect and the time × group interaction were obtained using a linear mixed-effects model. Time effect (*p*) refers to within-subject changes across the four assessment time points (baseline, week 4, week 8, week 12), whereas interaction effect (*p*) refers to the time × group interaction, indicating whether the pattern of change over time differed between vitamin D_3_ dose groups.

## Data Availability

The data that support the findings of this study are available from the corresponding author upon reasonable request.

## Abbreviations

The following abbreviations are used in this manuscript:

25(OH)D	25-Hydroxyvitamin D
ANOVA	Analysis Of Variance
APC	Article Processing Charge
BMI	Body Mass Index
BSAP	Bone-Specific Alkaline Phosphatase
CONSORT	Consolidated Standards of Reporting Trials
CTRI	Clinical Trials Registry of India
DAC	Doctoral Advisory Committee
DEXA	Dual-Energy X-Ray Absorptiometry
EAR	Estimated Average Requirement
FSSAI	Food Safety and Standards Authority of India
IEC	Institutional Ethics Committee
IOM	Institute Of Medicine
IU	International Units
PTH	Parathyroid Hormone
RCT	Randomized Controlled Trial
RDA	Recommended Dietary Allowance
SD	Standard Deviation
SPF	Sun Protection Factor
SPSS	Statistical Package for the Social Sciences
USDA	United States Department of Agriculture
VDR	Vitamin D Receptor

## Appendix A

**Table A1:** Baseline and Endline Diet nutrient and food group intake data by dose group.

Nutrient(s)	Timepoint	0 IU (*n* = 27)	400 IU (*n* = 26)	600 IU (*n* = 27)	800 IU (*n* = 26)
Energy (kcal)	Baseline	1536.72 ± 388.92	1664.03 ± 337.82	1680.72 ± 299.87	1554.74 ± 387.91
	Endline	1789.54 ± 326.60 *	1950.40 ± 500.71 *	1848.65 ± 405.31 *	1806.48 ± 444.89 *
Protein (g)	Baseline	51.51 ± 15.35	58.59 ± 14.16	56.70 ± 11.08	52.17 ± 13.39
	Endline	55.86 ± 14.69	61.65 ± 15.51	57.80 ± 14.78	60.11 ± 17.33 *
Fat (g)	Baseline	51.90 ± 18.47	54.51 ± 15.62	57.22 ± 15.03	49.84 ± 18.72
	Endline	63.07 ± 17.38 *	70.65 ± 24.82 *	67.11 ± 20.97 *	62.97 ± 21.65 *
Carbohydrate (g)	Baseline	216.93 ± 48.96	235.65 ± 47.10	235.69 ± 40.54	225.21 ± 48.96
	Endline	250.86 ± 36.21 *	268.27 ± 62.47 *	254.46 ± 49.87 *	250.95 ± 50.83 *
Calcium (mg)	Baseline	500.13 ± 178.51	483.07 ± 136.04	526.38 ± 138.80	495.05 ± 142.02
	Endline	629.65 ± 192.46 *	635.23 ± 199.68 *	619.72 ± 182.56 *	633.29 ± 211.15 *
Phosphorus (mg)	Baseline	1060.23 ± 281.52	1138.00 ± 226.52	1127.54 ± 190.73	1091.81 ± 289.97
	Endline	1205.58 ± 267.92 *	1283.21 ± 269.41 *	1224.74 ± 265.60 *	1229.67 ± 306.47 *
Iron (mg)	Baseline	12.45 ± 3.41	12.20 ± 3.53	12.86 ± 2.83	12.31 ± 3.39
	Endline	13.71 ± 3.57	13.78 ± 3.59	12.97 ± 3.15	12.74 ± 3.66

* Values are presented as Mean ± SD derived from 3-day dietary recall data at baseline and endline. Within group comparisons were assessed using the Wilcoxon Signed Ranks Test; Between-group differences at baseline and endline were evaluated using the Kruskal–Wallis Test and were not statistically significant (*p* > 0.05). A *p*-value < 0.05 indicates a significant within-group change. Nutrient intakes are categorized by intervention group (0 IU, 400 IU, 600 IU, and 800 IU vitamin D_3_/day).
